# Visual feedback adaptation enhances arm-posture coordination during floor-surface perturbations

**DOI:** 10.3389/fnhum.2025.1699598

**Published:** 2025-11-28

**Authors:** Yosuke Tomita, Hiroki Mani, Naoya Hasegawa

**Affiliations:** 1 Department of Physical Therapy, Faculty of Health Care, Takasaki University of Health and Welfare, Gunma, Japan; 2 Faculty of Welfare and Health Science, Oita University, Oita, Japan; 3 Department of Rehabilitation Science, Hokkaido University, Sapporo, Hokkaido, Japan

**Keywords:** postural control, sensorimotor integration, visual feedback, arm-posture coordination, perturbation, interjoint coordination

## Abstract

**Background:**

Maintaining postural stability during perturbations requires coordinated sensorimotor and interjoint coordination. This study investigated the effects of different feedback modalities (knowledge of results [KR] and continuous visual feedback) on postural adaptation during floor surface perturbations while standing.

**Methods:**

Nineteen healthy young adults (mean age: 23.1 ± 1.2 years; 12 males) performed an arm-holding task while standing on a backward-translating force platform under five phases: baseline test, KR adaptation training, post-KR adaptation (P-KRA) test, visual adaptation training, and post-visual adaptation (P-VA) test. Endpoint position variability, center of pressure (COP), center of mass (COM), margin of stability (MOS), and interjoint coordination were compared among Baseline, P-KRA, and P-VA using a mixed-model repeated-measures analysis of variance.

**Results:**

Compared to Baseline, endpoint position variability was significantly reduced in the P-VA at both perturbation offset (8.97 ± 1.04 mm vs. 15.35 ± 1.52 mm, *p* = 0.006) and 1.5 s after offset (14.39 ± 1.02 mm vs. 19.73 ± 1.71 mm, *p* = 0.027). The MOS at 1.5 s after offset was lower in P-VA (39.33 ± 4.28 mm) than in Baseline (58.04 ± 4.53 mm, *p* = 0.011), and the minimum MOS was significantly smaller in P-VA (32.20 ± 4.38 mm vs. 50.59 ± 4.26 mm, *p* = 0.011). Anticipatory COP displacement at onset in P-VA was significantly increased (14.11 ± 1.46 mm vs. 6.20 ± 0.89 mm, *p* < 0.001) and reduced peak forward COP displacement (89.42 ± 2.00 mm vs. 110.18 ± 3.35 mm, *p* < 0.001). The time to stability was shorter in P-VA (1,266.42 ± 68.29 ms) than in Baseline (1,525.78 ± 66.11 ms, *p* = 0.017). The cross-correlation coefficient between the elbow and ankle joints was significantly higher in P-VA than in Baseline (0.98 ± 0.01 vs. 0.89 ± 0.04, *p* = 0.014).

**Conclusion:**

These findings demonstrate that continuous visual feedback adaptation may enhance arm-posture coordination during external perturbations in healthy young adults.

## 1 Introduction

Postural stability requires continuous integration of proprioceptive, vestibular, and visual inputs to maintain body position and orientation during voluntary movements and external perturbations. This multisensory integration enables the adaptive reweighting of sensory signals, which is critical for populations with reduced mobility owing to motor and sensory impairments (Appiah-Kubi and Wright, 2019; Bugnariu and Fung, 2007; Liu et al., 2024). Studies have demonstrated that aging and neurological disorders disrupt sensory reweighting mechanisms, leading to increased reliance on visual feedback during balance tasks (Mahoney et al., 2019; Tran et al., 2023). While spinal reflexes (short-latency, < 50 ms) and brainstem-mediated vestibulospinal responses (mid-latency, 50–150 ms) provide rapid postural corrections, cortical pathways govern long-latency adjustments (>150 ms) through voluntary control mechanisms (Mochizuki et al., 2008; Takakusaki, 2017). These hierarchical neural processes are further modulated by anticipatory postural adjustments (APAs) when perturbations are predictable, thereby stabilizing the body through the feedforward control of the center of pressure (COP) trajectories (Chen et al., 2015; Hasegawa et al., 2021).

Arm–posture coordination is a critical component of human motor control, as maintaining stability often requires the simultaneous regulation of upper-limb and whole-body movements. Everyday activities, such as carrying a tray, holding an object, or stabilizing the hand in space while encountering balance disturbances, highlight the functional importance of hand-stabilizing tasks. These tasks increase coordination demands by requiring postural adjustments to support precise arm positioning, thereby providing a sensitive framework for investigating how voluntary upper-limb movements interact with balance responses. Compared with a stable seated position, standing increases the demand for postural adjustments owing to a smaller base of support and higher COM position (Mochizuki et al., 2024). Additionally, arm movements serve as critical compensatory mechanisms during significant balance threats, further intensifying the coordination demands between postural adjustments and voluntary arm movements (Maki and McIlroy, 1997; Pijnappels et al., 2010; Marigold and Misiaszek, 2009). Hand-stabilizing tasks highlight the need for effective sensorimotor adaptation mechanisms, particularly when balance must be maintained under perturbations. In the present study, arm-posture coordination was conceptualized as the concurrent regulation of upper-limb motion and postural stability during externally induced perturbations. This ability was quantified as the temporal coupling between the elbow and ankle joint movements, which represent the distal joints contributing to arm stabilization and postural recovery, respectively. A higher zero-lag cross-correlation coefficient between these joints indicates greater synchronization and, consequently, more efficient integration of postural and voluntary controls. This approach builds on our previous findings (Tomita et al., 2017a, 2023), which showed that effective arm–posture coordination can be maintained under increased postural demands, and extends this framework to examine how distinct visual feedback modalities influence such coordination during perturbation-based tasks.

Feedback-based adaptation plays a pivotal role in improving postural adjustment, with knowledge of results (KR) and continuous visual feedback representing distinct learning frameworks. KR enhances error detection by providing discrete summary information about performance outcomes after each movement, prompting participants to consciously reflect on their errors and adjust their subsequent movements accordingly (Winstein, 1991; Ronsse et al., 2011). In contrast, continuous visual feedback offers ongoing real-time spatial information that enables immediate online corrections of movement trajectories, potentially facilitating automatic implicit adjustments in postural control (Sigrist et al., 2013a,b). Previous studies have suggested that continuous visual feedback can rapidly reduce movement variability and improve accuracy in dynamic tasks (Vidal and Lacquaniti, 2021), whereas KR typically induces slower but potentially more robust adaptations via explicit cognitive processing (Ronsse et al., 2011). Although many studies have investigated KR and continuous visual feedback in seated or simplified upper-limb tasks (Ronsse et al., 2011; Vidal and Lacquaniti, 2021), relatively few have explored these modalities in conditions requiring active postural control. Studies using seated eyes-closed perturbation paradigms have shown that adaptive upper-limb responses to external perturbations depend on vestibular contributions (Sibindi et al., 2013; Raptis et al., 2007), suggesting that sensory integration mechanisms may differ when visual input is limited. In contrast, research on standing balance has demonstrated that real-time visual feedback of the COP enhances adaptive postural control (Takeda et al., 2017; Hasegawa et al., 2021). The present study extends this line of research by directly comparing discrete and continuous feedback modalities during a hand-stabilizing task performed under floor surface perturbations, providing new insights into how different feedback modalities shape postural and upper-limb coordination under dynamic balance demands. Therefore, this study aimed to examine the effects of different feedback modalities on postural adaptation to floor surface perturbations in healthy young adults. We hypothesized that continuous visual feedback would induce the most pronounced adaptive effects on postural control and coordination. Specifically, we expected that continuous visual feedback adaptation would reduce endpoint variability, increase dynamic postural stability, and increase interjoint coordination between ankle dorsiflexion and elbow flexion compared with other conditions.

## 2 Materials and methods

### 2.1 Participants

Nineteen healthy young adults with no history of neurological or musculoskeletal disorders participated in the study (mean ± standard deviation [min, max]: age, 23.1 ± 1.2 [21, 26] years; 12 males and 7 females; body weight, 58.7 ± 7.5 [42.3, 74.7] kg; height, 166.9 ± 7.8 [148.8, 182.7] cm). An a priori power analysis was conducted to estimate the required sample size. Assuming α = 0.05, correlation among repeated measures ρ = 0.50, and a large effect size (ηp^2^ ≥ 0.17; f = 0.45), the analysis indicated that a minimum of 19 participants would provide a statistical power of > 0.90. Participants completed a brief questionnaire regarding their medical history, including previous neurological and cardiovascular disorders. Individuals with such histories were excluded from the study. In addition, those who had experienced any injury that interfered with their daily activities within the past year or who reported current musculoskeletal pain (e.g., in the shoulder, back, or lower-limbs) that could affect their task performance were also excluded. In cases of uncertainty, eligibility was confirmed by a licensed physical therapist with over 10 years of clinical experience (N.H.) to ensure participant safety and minimize potential bias.

All participants provided written informed consent, and the study was approved by the Ethics Committee of the Faculty of Health Sciences, Hokkaido University (approval number: 23–19). This study was conducted in accordance with the principles of the Declaration of Helsinki. None of the participants had prior experience with experiments involving floor surface postural perturbation.

### 2.2 Experimental settings

Kinematic data were collected using a six-camera three-dimensional motion analysis system (Motion Analysis Corporation, Santa Rosa, CA, United States) with a sampling frequency of 200 Hz. Twenty-three reflective markers were attached to the following anatomical landmarks: sternum, bilateral frontal head, acromion, lateral epicondyle, radial head, tip of the index finger, anterior superior iliac spine (ASIS), greater trochanter, lateral femoral condyle, lateral malleolus, head of the second metatarsal, and calcaneus. One reflective marker was attached to the target, and two additional markers were placed on the force platform. These markers were used to calculate the COM in the anteroposterior direction using the head-arm-trunk model, which includes seven body segments: the upper body, thighs, shanks, and feet (Chang et al., 2017). This model assumes that the upper limbs move together with the trunk. Although the present task required the arms to maintain their position relative to the target, the arm posture remained nearly constant across all test phases. The participants stood on a force platform (Kistler, Winterthur, Switzerland; 74 × 74 × 10 cm) that was translated backward at a constant velocity of 66.0 cm/s triggered by a manual switch. The total translational displacement was 10.0 cm. Kinematic and kinetic data were collected at sampling frequencies of 200 Hz and 1,000 Hz, respectively. The platform returned to its original position after the end of each trial. This brief, controlled translation was designed to induce a transient mechanical disturbance in postural stability while maintaining a mechanically stable and rigid support surface. To ensure safety, two experimenters stood close to the participants to provide immediate manual support if necessary. No participant required assistance, and no adverse events occurred during the experiment.

### 2.3 Experimental procedures

**Figure 1:**
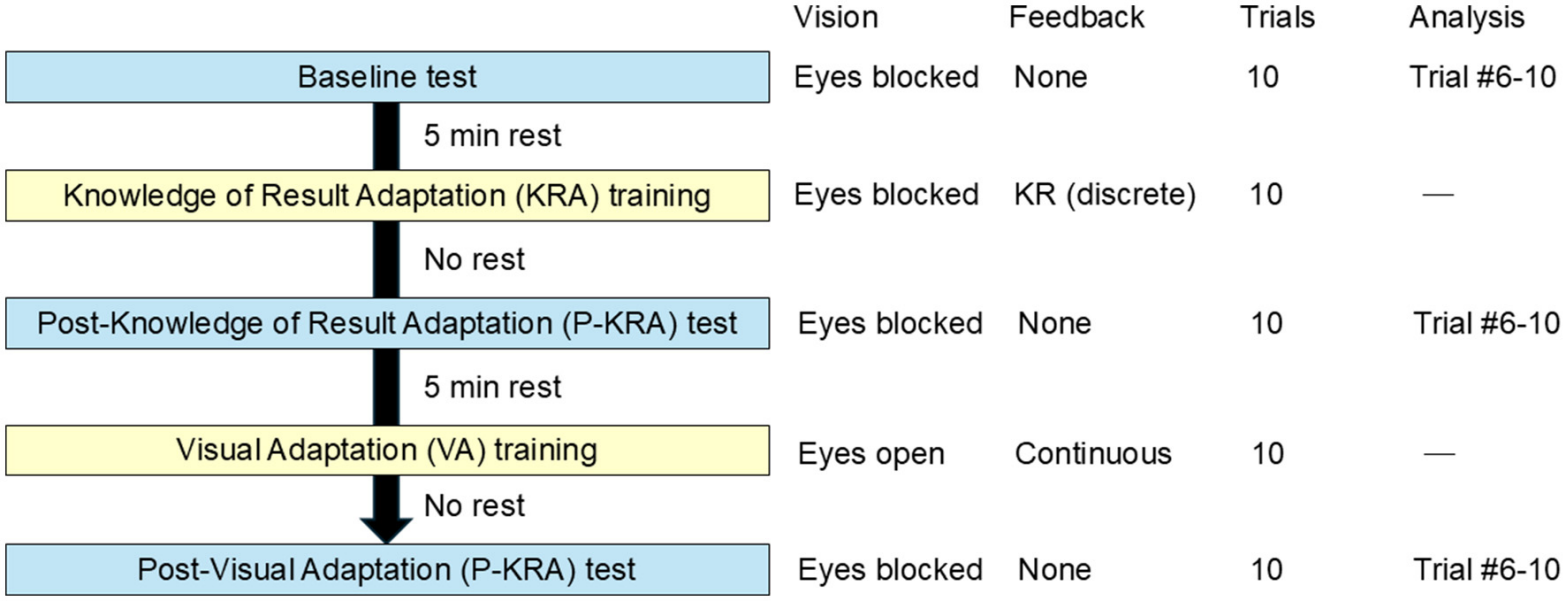
Experimental flow. Each participant completed 10 self-initiated perturbation trials per phase in the following fixed order: baseline test, Knowledge of Result Adaptation (KRA) training, post-KRA test, Visual Adaptation (VA) training, and post-Visual Adaptation (P-VA) test. Blue boxes represent test phases (Baseline, P-KRA, and P-VA), and yellow boxes represent training (adaptation) phases (KRA and VA). The vision and feedback conditions varied across phases: during Baseline, KRA, P-KRA, and P-VA, participants performed the task with eyes blocked, whereas the VA phase was performed with eyes open under continuous visual feedback. Each adaptation phase was immediately followed by its corresponding post-test phase (i.e., KRA to P-KRA and VA to P-VA) without a rest interval, while a 5-min seated rest was provided between other phases. Only the last five trials (Trials #6–#10) of each test phase were used for analysis to minimize the influence of early-trial variability.

The experiment consisted of five sequential phases, as illustrated in Figure 1. Each phase comprised 10 trials of a self-initiated postural perturbation task performed on a backward-translating force platform. The participants were instructed to maintain their fingertips as close as possible to a stationary target throughout each trial. The experiment consisted of five phases presented in a fixed order: Baseline test, KR adaptation (KRA) training, post-KR adaptation (P-KRA) test, visual adaptation (VA) training, and post-visual adaptation (P-VA) test. The KRA and VA phases served as training sessions, whereas the Baseline, P-KRA, and P-VA phases served as assessment sessions for statistical analyses. The last five trials (#6–#10) of each assessment phase were averaged to reduce the influence of early trial variability. All participants performed these phases in a fixed order to minimize the potential carryover from visual feedback to KR adaptation, as continuous visual feedback is known to induce a longer-lasting adaptation. Each training or assessment phase was separated by a 5-min seated rest period, except between the training and its subsequent post-assessment phases (i.e., KRA to P-KRA and VA to P-VA), which were performed consecutively. This fixed order was chosen because KR feedback provides discrete outcome-based information after each trial, whereas visual adaptation involves continuous real-time feedback. Randomizing these conditions could have caused residual effects from the visual feedback phase to carry over to the KR training and P-KRA test, thereby confounding the interpretation of each modality's effect. Previous studies have shown that discrete KR feedback is less susceptible to carryover than continuous feedback modalities (Hebert and Coker, 2021; Winstein and Schmidt, 1990). To reduce general learning effects, all participants completed a Baseline block prior to adaptation, and analyses were based on the mean of the last five trials of each adaptation phase.

**Figure 2:**
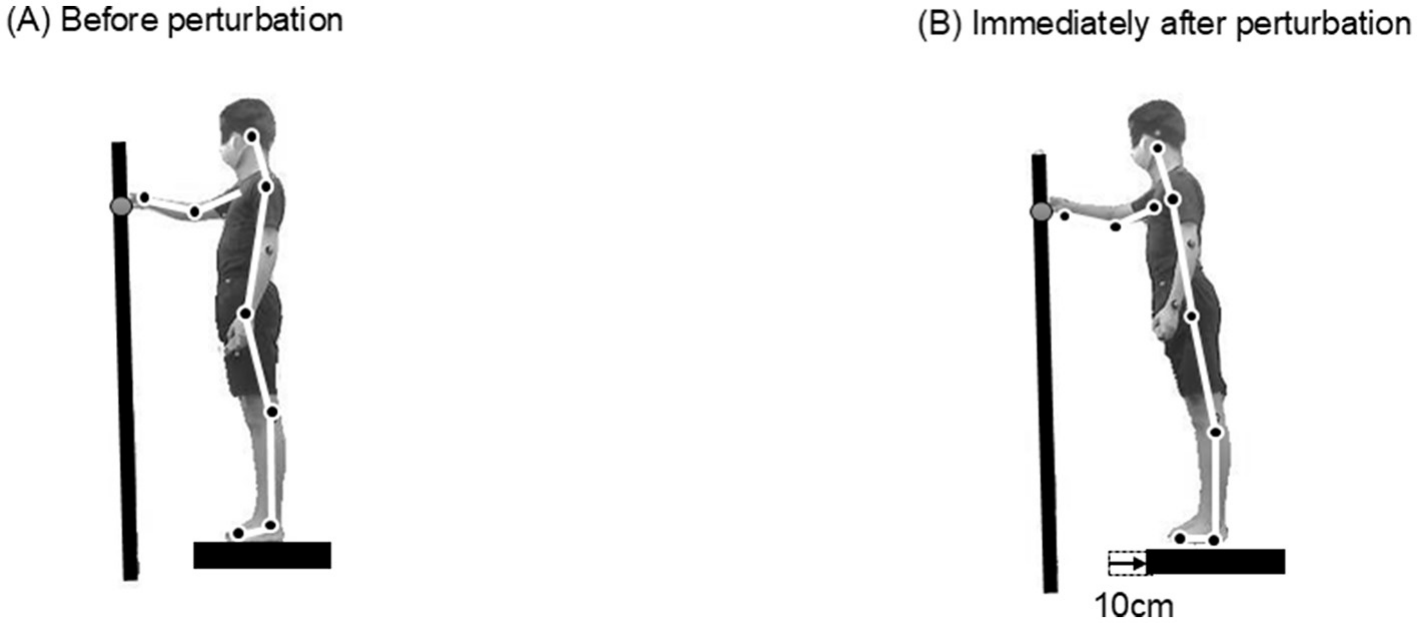
Experimental setup and task procedure. **(A)** Participants stood barefoot on a movable force platform with the tip of the dominant index finger positioned above a target. **(B)** Upon self-initiated backward translation of the platform (10 cm displacement at 66.0 cm/s), participants were instructed to maintain their index finger position above the target without stepping.

The target consisted of a reflective marker (2 cm in diameter) mounted on an adjustable stand. The target height was set at the participant's sternum level, and the anterior-posterior position was adjusted to match 2/3 of the length from the acromion to the radial head for each participant. This positioning ensured a consistent reaching distance across participants while maintaining a comfortable arm posture. In all conditions, the participants were instructed to maintain the fingertip position as close as possible to the target throughout the perturbation without stepping (Figure 2). Both aspects of the task were emphasized equally, and the participants were not instructed to prioritize either targeting accuracy or postural stability. Perturbations were self-initiated by pressing a handheld trigger with the non-dominant hand at a time of their choice. A trial was considered unsuccessful if a step was performed, and additional trials were conducted until 10 successful trials were completed.

During the Baseline test, the participants wore an eye mask and noise-canceling headphones, and no external feedback was provided. In the KRA training, vision was occluded during each trial, but after each trial, the eye mask was briefly removed so that participants could view the final finger position as discrete feedback. The P-KRA test was identical to the Baseline test and was conducted immediately after KRA training to evaluate the short-term retention of the KR-based adaptation training. In the VA training, the participants performed the task without an eye mask and received continuous visual feedback on their posture and endpoint position in real time throughout each trial. The P-VA test was identical to the Baseline test and was conducted immediately after VA training to assess the short-term retention of visual-feedback adaptation. Each condition consisted of 10 trials, and the participants rested for 5 min between conditions, with no additional washout period.

### 2.4 Visual feedback adaptation

The visual adaptation (VA) training utilized direct, unobstructed vision of the experimental environment, whereas the rest of the non-visual tests and trainings (Baseline, KRA, P-KRA, and P-VA) employed complete visual occlusion using an eye mask combined with noise-canceling headphones to eliminate both visual and auditory cues. In the VA training, participants performed the task with their eyes open, allowing natural vision of the task environment without artificial augmentation or modification. This direct vision provides continuous real-time visual feedback. No monitors, projections, or other visual display devices were used in the study. Visual feedback during VA training included three primary sources of information that participants could integrate simultaneously. First, the participants could directly observe the position of their index finger relative to the target throughout each trial, which enabled continuous monitoring of targeting accuracy. Second, they had full visual access to their body position and orientation in space, including trunk, limb, and head positions relative to the vertical. Third, the stationary laboratory environment, including a fixed target, provided stable external spatial references for both focal and postural control.

Participants were instructed to “look at the target and try to keep your finger as close to it as possible throughout the perturbation.” No additional gaze restrictions or specific gaze patterns were observed. Pilot observations confirmed that the participants predominantly maintained their gaze on the target during the trials, with occasional brief glances at other body parts or the environment.

Visual feedback during VA training was continuous throughout each trial, from the initial standing position through perturbation onset, during the perturbation (approximately 150 ms duration), and throughout the recovery phase until the participant achieved stability. The feedback was not interrupted, filtered, or modified at any point during the trials.

### 2.5 Data analysis

The marker position data were filtered using a fourth-order zero-lag low-pass Butterworth filter with a cutoff frequency of 20 Hz. The force platform was activated and stopped at the onset and offset of each perturbation, respectively. The following variables were computed: (1) endpoint metrics, (2) COM and COP metrics, and (3) angular metrics.

For the endpoint metrics, the displacement of the fingertip marker in the anteroposterior direction was calculated as both the mean change and standard deviation during two intervals: from perturbation onset to offset and from onset to 1.5 s after offset. Endpoint position variability was defined as the standard deviation of the fingertip position within each trial over the same intervals. The margin of stability (MOS) was calculated for the COM and COP metrics to assess postural stability. MOS was defined as the distance between the extrapolated COM (xCOM) and the anterior edge of the base of support in the sagittal plane. The xCOM was calculated according to Hof et al. (2005) using the following equation:


$$\begin{array}{l} xCOM=dCOM+\frac{vCOM}{\sqrt{g/l}} \end{array}$$ (1)

where *dCOM* is the COM displacement, *vCOM* is the COM velocity, *g* is the gravitational acceleration, and *l* is the distance from the ankle joint axis (lateral malleolus marker) to the COM in the sagittal plane. Because backward translation induces postural anterior instability, a toe marker was used to define the anterior boundary of the base of support. A higher MOS value indicates a more stable body configuration. The MOS was calculated at the onset, offset, and +1.5 s after offset, and the minimum MOS value during the trial was identified. For the COP displacement, the baseline COP position was computed by averaging the COP position between 1 and 3 s before the onset. The COP displacement was then measured relative to this baseline at the onset, offset, and 1.5 s after the offset. The peak COP displacements in the forward and backward directions were calculated separately. The time points corresponding to the minimum MOS, forward COP peak, and backward COP peak were identified. Time to stability was defined as the time at which the cumulative average of the anterior-posterior COP (COP_AP_) first fell within 0.25 standard deviations of the overall mean during the 3-s interval following the offset (Colby et al., 1999).

For the angular metrics, the joint angles were first calculated from the three-dimensional marker trajectories. The elbow flexion/extension angle was computed using the dot product of the upper arm and forearm segment vectors. For all other joints, the angles were determined as planar angles projected onto the sagittal plane, representing flexion/extension of the shoulder, hip, knee, and dorsiflexion/plantarflexion of the ankle. The following joint angles were computed at offset and +1.5 s after the offset: elbow flexion, shoulder flexion, shoulder horizontal flexion, hip flexion, trunk pitch, and ankle dorsiflexion. Interjoint coordination was evaluated using the zero-lag cross-correlation coefficient between the following joint pairs: shoulder flexion-ankle dorsiflexion and elbow flexion–ankle dorsiflexion.

The 1.5 s window for kinematic variables was used to evaluate the control process during the balance recovery phase, providing a standardized snapshot of postural behavior across conditions. In contrast, the 3.0 s window used for the TTS metric was designed to capture the total duration required to regain postural stability. These different time thresholds (i.e., 1.5 s for endpoint, MOS, and COP metrics, and 3.0 s for TTS) were selected to represent distinct but complementary aspects of postural adjustments after perturbation.

### 2.6 Statistical analysis

The mean values of the last five trials for each condition were calculated for all outcome measures. Data are presented as mean ± standard deviation. The independent variable was test phases, which included three levels corresponding to the analyzed datasets: Baseline, Post-KR adaptation (P-KRA), and Post-Visual adaptation (P-VA). The dependent variables included (1) endpoint metrics (endpoint displacement and position variability), (2) postural stability metrics (margin of stability [MOS], center of pressure [COP] displacement, and time-to-stability [TTS]), and (3) angular metrics (joint angles and cross-correlation coefficients). The KR and Visual adaptation phases were used as training sessions for feedback-based motor adaptation and were not included in the main statistical analyses. Only the three closed-eye test phases (Baseline, Post-KRA, and Post-VA) were compared to evaluate the transfer effects under identical visual conditions. A mixed-model repeated-measures analysis of variance with the factor test phases (three levels: Baseline, P-KRA, P-VA) was performed separately for each dependent variable. Effect sizes were calculated as partial η^2^ (ηp^2^). When significant main effects were observed in the mixed-model ANOVA, Bonferroni-corrected *post hoc* comparisons were performed among the three test phases (Baseline, P-KRA, and P-VA). A total of three pairwise comparisons were tested (Baseline vs. P-KRA, Baseline vs. P-VA, and P-KRA vs. P-VA), and the adjusted significance threshold was set at α = 0.017 (0.05/3). All *post hoc p*-values presented in the tables are Bonferroni-adjusted values. All statistical analyses were performed using SPSS ver. 21 (IBM Japan, Tokyo, Japan).

## 3 Results

### 3.1 Endpoint metrics

**Figure 3:**
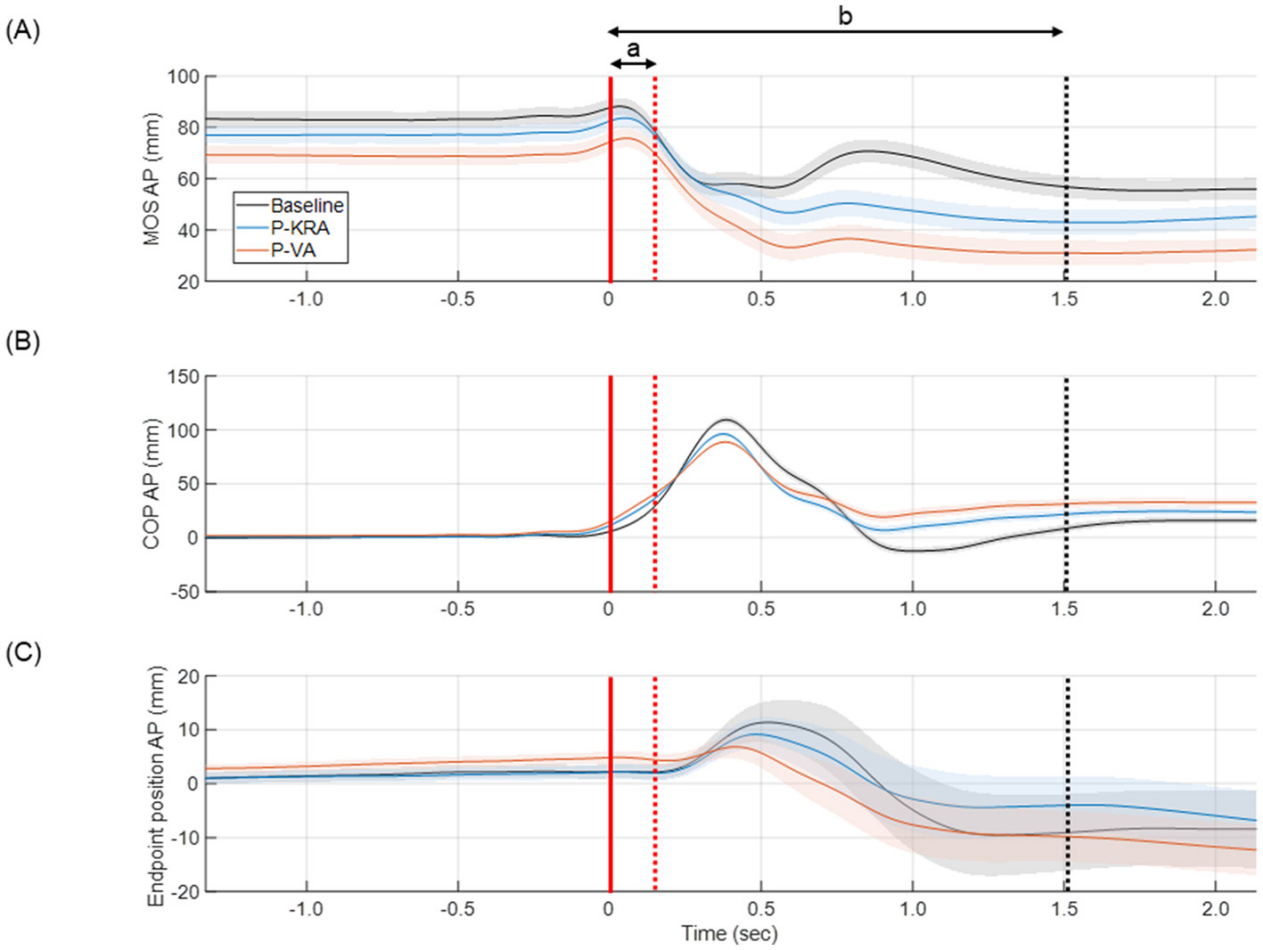
Condition-dependent time courses of MOS, COP-AP, and endpoint position aligned to perturbation onset. Mean (solid lines) ± 95% CI (shaded) time courses of **(A)** margin of stability in the anteroposterior direction (MOS), **(B)** center of pressure in the anteroposterior direction (COP-AP), and **(C)** endpoint (finger) position in the anteroposterior direction, all time-aligned to the perturbation onset (solid red line at 0 s). The dotted red line indicates perturbation offset (~0.15 s), and the dotted black line indicates +1.5 s after offset, which was the end of the analyzed recovery phase. Analysis intervals are denoted by “**a**” (perturbation phase: onset–offset) and “**b**” (recovery phase: offset–+1.5 s). Experimental conditions are color-coded as Baseline (black), P-KRA (blue), and P-VA (orange). The curves represent the averaged trajectories across all trials and participants.

The endpoint displacements were similar across all test phases (Figure 3, Table 1). However, significant differences in endpoint position variability were observed. At offset, the endpoint position variability was significantly lower in the P-VA than in the Baseline. At +1.5 s after offset, endpoint position variability was significantly reduced in both the P-KRA and P-VA compared to the Baseline.

**Table 1:** Endpoint metrics.

	**Baseline**	**P-KRA**	**P-VA**	***F*-value**	***P*-value**	**ηp^2^**
**Endpoint displacement (mm)**						
Offset	10.51 ± 4.33	6.18 ± 4.05	4.50 ± 3.48	0.596	0.556	0.032
+1.5 s offset	−8.76 ± 9.10	−8.17 ± 7.31	−11.59 ± 6.84	0.065	0.937	0.004
**Endpoint position variability (mm)**						
Offset	15.35 ± 1.52	11.09 ± 1.33	8.97 ± 1.04^*^	6.009	0.006	0.250
+1.5 s offset	19.73 ± 1.71	14.46 ± 1.09^*^	14.39 ± 1.02^*^	4.053	0.027	0.184

P-KRA, Post-Knowledge of Results Adaptation; P-VA, Post-Visual Adaptation.

^*^Significant difference compared to Baseline (*p* < 0.05, Bonferroni-corrected).

^†^Significant difference compared to P-KRA (*p* < 0.05, Bonferroni-corrected).

### 3.2 Center of mass (COM) and center of pressure (COP) metrics

For the MOS, there was a decreasing trend after adaptation, with the lowest values observed in the P-VA (Figure 3, Table 2). At offset, the MOS did not differ significantly among the test phases (F = 3.224, *p* = 0.051). However, at +1.5 s after offset, MOS was significantly smaller in P-VA than in the Baseline (*F* = 5.055, *p* = 0.011). The minimum MOS was significantly smaller in the P-VA than in the Baseline and in the P-KRA (*F* = 5.103, *p* = 0.011).

**Table 2:** COM and COP metrics.

	**Baseline**	**P-KRA**	**P-VA**	***F*-value**	***P*-value**	**ηp^2^**
**MOS (mm)**						
Offset	57.05 ± 4.61	55.48 ± 5.15	40.83 ± 5.09	3.224	0.051	0.152
+1.5 s after offset	58.04 ± 4.53	53.85 ± 4.52	39.33 ± 4.28^*^	5.055	0.011	0.219
Minimum MOS	50.59 ± 4.26	47.55 ± 4.60	32.20 ± 4.38^*†^	5.103	0.011	0.221
**COP displacement (mm)**						
Onset	6.20 ± 0.89	11.28 ± 1.22^*^	14.11 ± 1.46^*†^	12.681	< 0.001	0.413
Offset	77.66 ± 5.63	61.45 ± 4.96	63.30 ± 4.39	2.745	0.078	0.132
+1.5 s after offset	16.42 ± 3.81	20.16 ± 4.34	29.05 ± 4.49	2.346	0.109	0.115
Max COP	110.18 ± 3.35	98.03 ± 2.58^*^	89.42 ± 2.00^*†^	14.70	< 0.001	0.449
Min COP	−18.37 ± 4.09	−5.99 ± 3.73	1.55 ± 3.74^*†^	6.516	0.004	0.266
**Time metrics (ms)**						
Minimum MOS	860.88 ± 99.70	830.88 ± 77.09	823.42 ± 85.14	0.044	0.957	0.002
Forward COP peak	291.63 ± 4.17	285.50 ± 3.25	292.11 ± 2.79	1.312	0.283	0.068
Backward COP peak	954.13 ± 195.32	1,105.88 ± 324.86	1,461.27 ± 425.78	0.601	0.554	0.032
Time to stability	1,525.78 ± 66.11	1,317.22 ± 45.19^*^	1,266.42 ± 68.29^*†^	4.527	0.017	0.201

COM, Center of mass; COP, Center of pressure; MOS, Margin of stability; P-KRA, Post-Knowledge of Results Adaptation; P-VA, Post-Visual Adaptation.

^*^Significant difference compared to Baseline (*p* < 0.05, Bonferroni-corrected).

^†^Significant difference compared to P-KRA (*p* < 0.05, Bonferroni-corrected).

At perturbation onset, COP displacement was significantly larger in both P-KRA and P-VA than at Baseline, and it further increased in P-VA compared with P-KRA (*F* = 12.681, *p* < 0.001). However, at offset and +1.5 s after offset, no significant differences were observed among the test phases (offset: *F* = 2.745, *p* = 0.078; +1.5 s after offset: *F* = 2.346, *p* = 0.109). Regarding COP displacements, the maximum COP (i.e., peak forward displacement) was significantly smaller in both P-KRA and P-VA than in the Baseline, with a further reduction in P-VA relative to P-KRA (*F* = 14.70, *p* < 0.001). Conversely, the minimum COP (i.e., peak backward displacement) was also significantly smaller in P-VA than in both the Baseline and P-KRA (*F* = 6.516, *p* = 0.004), indicating reduced overall COP excursions following visual feedback adaptation.

For the time metrics, there were no significant differences in the timing of the minimum MOS, maximum COP, or minimum COP among the test phases. However, the time to stability was significantly shorter in both P-KRA and P-VA than in the Baseline, with a further reduction in P-VA relative to P-KRA (*F* = 4.527, *p* = 0.017), indicating faster postural recovery following visual feedback adaptation.

### 3.3 Angular metrics

No significant differences were observed among the test phases for elbow flexion, shoulder flexion, shoulder horizontal flexion, and ankle dorsiflexion angles at either offset or +1.5 s after offset (Table 3). The hip flexion angle showed significant differences across test phases. At offset, hip flexion was significantly smaller in P-VA compared with both the Baseline and P-KRA (*F* = 4.03, *p* = 0.027), and this reduction remained significant at +1.5 s after offset (*F* = 4.147, *p* = 0.024). The trunk pitch angle was also significantly smaller in P-VA than in both Baseline and P-KRA at offset (*F* = 4.402, *p* = 0.019) and +1.5 s after offset (*F* = 4.11, *p* = 0.024). No significant differences were observed in the ankle dorsiflexion angle at the offset (*F* = 0.783, *p* = 0.465) or at +1.5 s after the offset (*F* = 2.046, *p* = 0.143). The cross-correlation coefficientt between shoulder flexion and ankle dorsiflexion did not showany significant differences among the test phases. However, the cross-correlation coefficient between elbow flexion and ankle dorsiflexion was significantly higher in P-VA compared with both the Baseline and P-KRA (*F* = 5.224, *p* = 0.014).

**Table 3:** Angular metrics.

	**Baseline**	**P-KRA**	**P-VA**	***F*-value**	***P*-value**	**ηp^2^**
**Elbow flexion angle (deg)**						
Offset	2.36 ± 1.02	2.05 ± 0.84	2.07 ± 0.67	0.035	0.966	0.002
+1.5 s offset	0.37 ± 1.35	0.37 ± 1.16	1.03 ± 0.96	0.126	0.882	0.007
**Shoulder flexion angle (deg)**						
Offset	−1.74 ± 0.79	−1.44 ± 0.62	−1.91 ± 0.43	0.198	0.821	0.011
+1.5 s offset	−0.79 ± 1.11	−1.38 ± 1.07	−2.53 ± 0.71	1.019	0.371	0.054
**Shoulder horizontal flexion angle (deg)**						
Offset	1.76 ± 2.24	0.40 ± 2.39	0.50 ± 1.80	0.120	0.887	0.007
+1.5 s offset	4.43 ± 3.05	2.02 ± 3.26	1.25 ± 2.88	0.305	0.739	0.017
**Hip flexion angle (deg)**						
Offset	−2.50 ± 0.420	−3.32 ± 0.34	−4.00 ± 0.33^*^	4.03	0.027	0.183
+1.5 s offset	0.64 ± 0.73	−0.140 ± 0.69	−1.72 ± 0.50^*^	4.147	0.024	0.187
**Trunk pitch angle (deg)**						
Offset	12.29 ± 1.11	10.38 ± 1.15	8.15 ± 0.88^*†^	4.402	0.019	0.197
+1.5 s offset	18.81 ± 1.44	17.14 ± 1.65	13.31 ± 1.34^*†^	4.11	0.024	0.186
**Ankle dorsiflexion angle (deg)**						
Offset	4.96 ± 0.86	4.03 ± 0.68	5.17 ± 0.68	0.783	0.465	0.042
+1.5 s offset	−3.99 ± 0.84	−3.13 ± 0.99	−1.71 ± 0.78	2.046	0.143	0.102
**Cross-correlation coefficient**						
Shoulder flexion-ankle dorsiflexion	−0.28 ± 0.17	−0.25 ± 0.20	−0.27 ± 0.19	0.007	0.993	0.000
Elbow flexion ankle-ankle dorsiflexion	0.89 ± 0.04	0.94 ± 0.01	0.98 ± 0.01^*†^	5.224	0.014	0.225

P-KRA, Post-Knowledge of Results Adaptation; P-VA, Post-Visual Adaptation.

^*^Significant difference compared to Baseline (*p* < 0.05, Bonferroni-corrected).

^†^Significant difference compared to P-KRA (*p* < 0.05, Bonferroni-corrected).

## 4 Discussion

This study aimed to evaluate the effects of different types of feedback adaptation on postural adjustment in response to perturbations in the floor surface. Endpoint displacement (i.e., accuracy) remained consistent across test phases, whereas endpoint position variability (i.e., precision) was significantly lower in the P-VA test than in the Baseline at offset and reduced in both P-KRA and P-VA at +1.5 s after offset. MOS decreased following adaptations, with the lowest values observed in P-VA; MOS at +1.5 s after offset was significantly smaller in P-VA than at Baseline, and the minimum MOS was significantly smaller in P-VA than in both Baseline and P-KRA. COP displacement at onset was significantly larger in both P-KRA and P-VA than at Baseline, with the largest displacement observed in P-VA. The maximum and minimum COP displacements were significantly smaller in P-KRA and P-VA, with further reductions observed in P-VA compared with P-KRA. Hip flexion and trunk pitch angles were smaller in P-VA compared with Baseline, and the trunk pitch angle was further reduced compared with P-KRA. The cross-correlation coefficient between elbow flexion and ankle dorsiflexion was significantly higher in P-VA than in both the Baseline and P-KRA. These findings suggest that continuous visual feedback adaptation may enhance arm-posture coordination.

### 4.1 Enhanced movement precision through continuous visual feedback adaptation

The significant reduction in endpoint position variability observed in the P-VA at both offset and +1.5 s after the offset suggests that visual feedback enhances movement precision via distinct neural mechanisms. The increase in endpoint position variability at +1.5 s after the offset, compared to that at the offset, may be attributed to the engagement of different neural processes in arm-posture adjustments at these time points. Specifically, the period immediately following the perturbation offset (within approximately 150 ms) predominantly involves mid-latency responses such as vestibulospinal reflexes, which are automatic and rapid reactions mediated by subcortical structures, including the brainstem and spinal circuits (Rosengren and Colebatch, 2002). In contrast, adjustments observed at +1.5 s after the offset are likely governed by late-latency responses involving voluntary control from the motor cortex, reflecting more deliberate and conscious postural corrections (Jacobs and Horak, 2007). The increased variability during these voluntary responses may arise from the inherent flexibility and adaptability of cortical control mechanisms, which, while enabling nuanced adjustments, can introduce greater variability than the stereotyped responses of subcortical reflex pathways. Notably, the present study observed differential effects between P-KRA and P-VA, particularly in postural adjustment-related variables, such as anticipatory COP displacement, maximum and minimum COP excursions, and trunk pitch angle. These differences suggest that continuous visual feedback adaptation may modulate cortical control processes governing postural adjustments more effectively than discrete knowledge-of-results feedback. This interpretation aligns with evidence that continuous visual feedback facilitates online error correction and promotes refined sensorimotor recalibration during balance tasks (Sigrist et al., 2013a,b; Takeda et al., 2017).

The KRA training phase provided discrete, outcome-based feedback that promoted explicit learning through conscious error evaluation and strategic adjustment. This interpretation aligns with findings by Hebert and Coker (2021), which demonstrated that moderate or self-controlled KR frequency optimizes motor learning by intrinsic error detection. In contrast, continuous visual feedback provides real-time, ongoing sensory information that supports implicit sensorimotor recalibration through automatic error correction during the execution of a task. Notably, continuous feedback tends to reduce cognitive load during movement and facilitates the acquisition of more stable and generalized motor patterns, as shown in prior studies on concurrent feedback training (Wulf and Shea, 2002; Sigrist et al., 2013a,b). This reduced attentional demand enables the efficient integration of multimodal sensory inputs and supports the development of automatic, feedback-driven adjustments. Conversely, discrete KR feedback stabilizes performance outcomes but may maintain greater variability in movement patterns, reflecting trial-by-trial exploration inherent to explicit learning (Salmoni et al., 1984). Furthermore, studies have shown that KR-based learning primarily recruits cortico-striatal networks associated with reinforcement learning, whereas feedback-error-driven adaptation relies more on cerebellar circuits (Bostan and Strick, 2018; Doyon et al., 2009). In this context, the present results, showing larger anticipatory COP displacement, smaller trunk and hip flexion angles, and stronger ankle–elbow coupling in the P-VA test phase, suggest that continuous visual feedback facilitates implicit postural adjustments, consistent with cerebellar-mediated error-based learning. In contrast, the P-KRA, which provided discrete, outcome-based information after each trial, likely promoted consciously mediated postural regulation through explicit reinforcement processes engaging the basal ganglia-cortical circuits.

Taken together, these results support the notion that visual feedback adaptation and KR adaptation may engage distinct neural learning systems. This differentiation is consistent with theoretical models of motor learning, suggesting that concurrent feedback (i.e., VA) minimizes cognitive load and stabilizes general movement patterns (Wulf and Shea, 2002; Sigrist et al., 2013a,b), whereas discrete KR feedback (i.e., KRA) enhances explicit error evaluation but retains greater variability in movement execution (Salmoni et al., 1984).

### 4.2 Enhanced postural adjustments accompanied by increased interjoint coupling between ankle and elbow joints

Contrary to our original hypothesis, the minimum MOS in the P-VA test was significantly lower than that in the Baseline and P-KRA tests. Rather than indicating enhanced postural stability, this reduction in the MOS suggests a shift in the postural strategy under continuous visual feedback. Specifically, a smaller MOS may reflect a more forward COM excursion relative to the base of support—i.e., participants may have accepted narrower safety margins in favor of tighter coupling between postural and focal task performance. Such precise regulation of the COM position may result from anticipatory COP adjustments at the perturbation onset (Hasegawa et al., 2021), enabling the system to manage the COM position with reduced forward COP displacement during the perturbation response. Consequently, this mechanism led to a diminished subsequent backward sway in the P-VA compared with the Baseline. The observed reductions in hip flexion and trunk pitch angles indicate that continuous visual feedback adaptation promotes a more upright posture. This finding aligns with biomechanical principles, as a more upright posture reduces the anterior body weight shift, thereby decreasing hip flexion torque and the demand for hip extensor muscles (Masani et al., 2013). This shift toward distal control reduces the reliance on hip-based stabilization strategies and promotes a more distributed control mechanism that integrates proximal and distal joint contributions to maintain balance (Morasso, 2022). Importantly, unlike typical postural perturbation tasks, where upper limb movements primarily assist in balance recovery (Pijnappels et al., 2010), the present task required the upper limb to remain positioned over the target despite perturbations. This task demand necessitated a higher level of interjoint coordination, where postural adjustments had to be synchronized with upper limb stability. Qu et al. (2024) found that individuals who successfully recovered from trip-induced perturbations exhibited greater coupling between lower limb joints, particularly between the knee and ankle joints, whereas those who failed to regain balance demonstrated increased variability in distal joint movements. Furthermore, perturbation-based training has been shown to induce changes in muscle synergies among lower limb muscles (Wang et al., 2022). Our study extends these findings by demonstrating that the temporal coupling between the upper-limb (elbow) and lower-limb (ankle) also increases following continuous visual feedback. This strategy aligns with the findings that effective coordination between the distal and proximal joints contributes to maintaining balance during perturbations (Boström et al., 2018). Furthermore, in the P-VA, COP displacement at perturbation onset was the largest, whereas the maximum COP excursion after perturbation was reduced compared with the Baseline. This pattern suggests that the participants performed greater anticipatory COP adjustments before perturbation onset, enabling them to minimize reactive COP displacement during the balance recovery phase. The preparatory shift in the COP may reflect an enhanced ability to prepare for external perturbations under continuous visual feedback.

In this study, coordination is defined, following Tomita et al. (2017b), as “a goal-oriented process in which degrees of freedom are organized in both spatial and temporal domains such that the body configuration enables the endpoint to reach a desired location in a context-dependent manner”. In our dual-task context, participants were required to maintain their COM position within their BOS while simultaneously keeping their fingertip on a stationary target during postural perturbations. Therefore, arm-posture coordination in our study represented the process by which the body dynamically organized postural and upper-limb motions to achieve both postural stability and focal precision goals. We interpret the “enhanced arm–posture coordination” observed in this study as a refinement of this organizational process, in which both postural and focal task performances become more efficiently integrated through a strengthened coupling between segments. In the current study, this refinement was reflected in (a) increased anticipatory postural adjustments (i.e., COP displacements before perturbation onset) and (b) stronger temporal coupling between the ankle and elbow joints following visual adaptation, suggesting a more coherent organization of postural and focal adjustments. Therefore, our results suggest that continuous visual feedback adaptation facilitates a more integrated form of arm–posture coordination.

### 4.3 Limitations

One limitation of this study was the absence of retention tests, which restricts the understanding of the long-term effects of feedback-based adaptation on the coordination between postural adjustments and arm movements. Without assessing whether changes persist beyond the immediate adaptation phase, it remains unclear whether the observed effects reflect lasting benefits or merely short-term adjustments, such as electromyography, motor-evoked potentials (MEPs), or H-reflex responses, which could offer direct insights into the neural mechanisms underlying the observed behavioral adaptations. Incorporating such assessments would help clarify how feedback modalities influence sensorimotor integration during postural perturbations and further elucidate the differential contributions of cortical and subcortical pathways to feedback-based learning. Future research should include both retention assessments and neurophysiological measures to comprehensively evaluate the durability and neural basis of the observed adaptations. Another limitation is related to the fixed order of the experimental phases. Although this sequence was intentionally adopted to minimize the carryover effects from visual feedback to the KR, the fixed order may have introduced cumulative or temporal practice effects that cannot be fully ruled out. Therefore, changes observed in the later phases might partially reflect general learning or sequence-dependent influences rather than feedback-specific adaptation. Future studies should consider counterbalancing or randomizing the order of feedback conditions and including washout phases to better isolate modality-specific effects. Another potential limitation of the present study is that visual information was completely occluded during non-visual conditions. Although healthy young adults can maintain postural stability by increasing the relative weighting of vestibular and somatosensory inputs when vision is unavailable (Peterka, 2002), the influence of full visual occlusion on balance control cannot be entirely ruled out. In populations such as older adults or individuals with neurological disorders, who rely more heavily on visual input for postural regulation, the present design may not be appropriate. Future studies should consider selectively removing only target-related visual information, for example, by using a screen-based target that disappears, to minimize potential interference with postural control. Although the present study results may provide insight into the potential mechanisms relevant to populations with impaired sensory integration, such as older adults or individuals with neurological disorders, caution is warranted when extrapolating the current findings. Future studies are needed to verify whether similar changes occur after continuous visual feedback adaptation in clinical populations and to examine the extent to which these mechanisms contribute to enhancing arm–posture coordination in these groups.

## 5 Conclusions

This study examined the effects of different feedback modalities on postural adaptation to floor surface perturbations in healthy young adults. Nineteen healthy young adults performed perturbation tasks under discrete knowledge-of-results and continuous visual feedback conditions. Continuous visual feedback led to reduced endpoint variability, smaller trunk and hip angles, and stronger ankle–elbow joint coupling, indicating enhanced integration of postural and focal adjustments. These findings suggest that continuous visual feedback promotes implicit adaptation of arm–posture coordination.

## Data Availability

The raw data supporting the conclusions of this article will be made available by the authors, without undue reservation.
